# Oral interactions between a green tea flavanol extract and red wine anthocyanin extract using a new cell-based model: insights on the effect of different oral epithelia

**DOI:** 10.1038/s41598-020-69531-9

**Published:** 2020-07-28

**Authors:** Susana Soares, Sónia Soares, Elsa Brandão, Carlos Guerreiro, Nuno Mateus, Victor de Freitas

**Affiliations:** LAQV/REQUIMTE, Faculdade de Ciências da Universidade Do Porto, Rua Do Campo Alegre, 687, 4169-007 Porto, Portugal

**Keywords:** Natural products, Nutrition

## Abstract

Phenolic compounds (PC) are linked to astringency sensation. Astringency studies typically use simple models, with pure PC and/or proteins, far from what is likely to occur in the oral cavity. Different oral models have been developed here, comprising different oral epithelia (buccal mucosa (TR146) and tongue (HSC-3)) and other main oral constituents (human saliva and mucosal pellicle). These models, were used to study the interaction with two PC extracts, one rich in flavanols (a green tea extract) and one rich in anthocyanins (a red wine extract). It was observed that within a family of PC, the PC seem to have a similar binding to both TR146 and HSC-3 cell lines. When the oral constituents occur altogether, flavanols showed a higher interaction, driven by the salivary proteins. Conversely, anthocyanins showed a lower interaction when the oral constituents occur altogether, having a higher interaction only with oral cells. Epigallocatechin gallate, epicatechin gallate, epigallocatechin-3-*O*(3-*O*-methyl) gallate were the flavanols with the highest interaction. For the studied anthocyanins (delphinidin-3-glucoside, peonidin-3-glucoside, petunidin-3-glucoside and malvidin-3-glucoside), there was not a marked difference on their interaction ability. Overall, the results support that the different oral constituents can have a different function at different phases of food (PC) intake. These differences can be related to the perception of different astringency sub-qualities.

## Introduction

Phenolic compounds have long been known for their health benefits, in particular cardiovascular protection, and antioxidant activity. Their importance goes beyond the health benefits, since some phenolic compounds are linked to the organoleptic properties of plant-based foodstuffs, namely color, astringency and bitterness.

Color is one of the first assessed quality parameter and in some cases one of the most important for consumer acceptance. A depreciative visual perception could lead to an immediately rejection of a product without any further evaluation of the consumer. Astringency and bitter taste are hand-in-hand taste properties of plant-based foodstuffs frequently perceived as unpleasant. In fact, several approaches are used to disguise these unpleasant taste properties, like adding sugar to decrease bitterness^1^, or adding milk to decrease tea astringency^1^.

The physiological mechanisms for the perception of these taste properties are quite different. The perception of bitter taste is well-characterized and occurs through activation of specific membrane G protein coupled receptors, the bitter taste receptors (TAS2Rs)^2^. Astringency is described as a tactile sensation of puckering, roughness and constriction in the oral cavity. Contrasting to bitterness, the molecular perception of astringency has been a thoroughly debated topic^3–6^. One of the most important mechanisms, is based on the interaction and precipitation of salivary proteins, in particular proline-rich proteins (PRPs), by food tannins.

Among the most important families of salivary proteins related to astringency are the different classes of PRPs, mucins, statherin and P-B peptide. PRPs are classically divided into basic, glycosylated and acidic PRPs^7^. Structurally these classes have only few differences: basic PRPs are the richest in proline residues and also in glycine residues, which together account for 70% of their sequence^7^; glycosylated PRPs present carbohydrates linked to some amino acid residues ^7^; and, acidic PRPs are rich in acidic amino acid residues, such as glutamic acid (or glutamine)^7^. Mucins are a family of largely glycosylated proteins with high molecular weight and they are the main components of mucous. The salivary mucins are MUC5B, MUC7, MUC19, MUC1 and MUC4 and among these MUC5B is thought to be the predominant gel-forming mucin in the oral cavity^8^. Statherin is an acidic salivary protein rich in tyrosine and its function is to maintain the homeostasis of calcium phosphate in saliva, needed for the remineralization of tooth enamel^9^. P-B peptide is generally included into the basic PRPs family due to its very high content in proline residues (50% of its sequence) but in fact it displays some structural similarities with statherin^10^.

Over the last years, strong data have been gathered supporting that, despite some phenolic compounds precipitate indeed salivary proteins, this mechanism does not explain the astringency of all compounds. Some astringent compounds have proved to fail to precipitate salivary proteins. Also, an additional reported point is that astringency can be perceived in different oral tissues, both in non-gustatory (lips) as well as in gustatory (tongue) oral epithelia. In fact, other hypothesis have been raised, such as the activation of mechanoreceptors, the involvement of oral cells^11,12^ and/or the involvement of the mucosal pellicle^13^. Although no solid evidence has been proved regarding the activation of mechanoreceptors, it has been already shown that astringency is in fact perceived by activation of the trigeminal ganglion nerve^14^. Regarding the involvement of oral cells and mucosal pellicle on astringency, several reports have shown that both these oral components can bind to phenolic compounds and therefore could have an active role in astringency perception.

**Figure 1:**
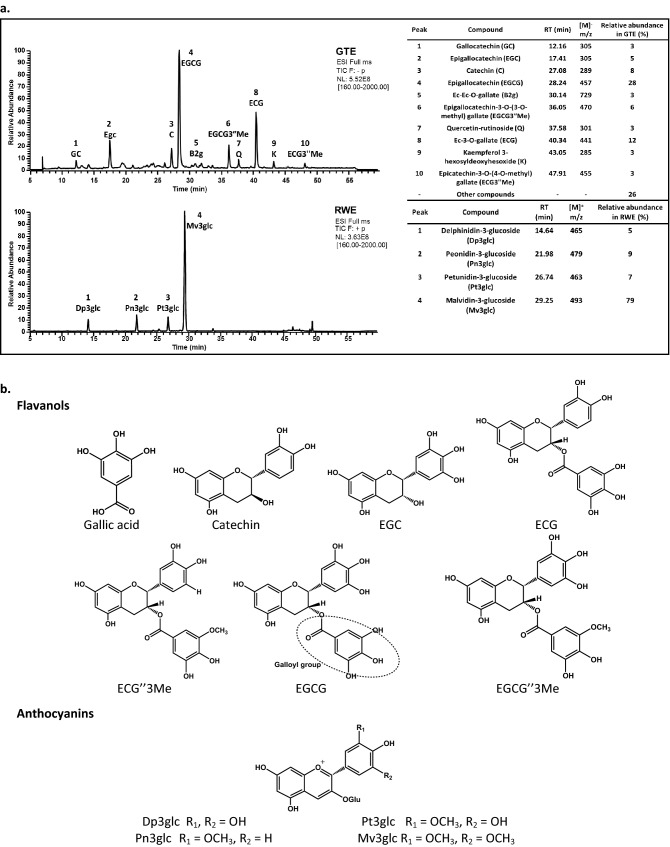
(**a**) Total ion current (TIC) profile of the proanthocyanidins green tea extract (GTE) at 1.0 mg mL^−1^ and of the anthocyanins red wine extract (RWE) at 0.6 mg mL^−1^ obtained by LC–MS analysis. The table presents the identity of each peak as well as the relative proportion of each compound to the whole mixture. (**b**) The structure of the identified phenolic compounds divided into their familis: flavanols and anthocyanins. *EGC* epigallocatechin gallate; *ECG* epicatechin gallate, *ECG″3Me*, epicatechin-3-*O*-(3-*O*-methyl) gallate; *EGCG* epigallocatechin gallate; *EGCG″3Me*, epigallocatechin-3-*O*-(3-*O*-methyl) gallate; *DP3glc* delphinidin-3-glucoside; *Pn3glc* peonidin-3-glucoside; *Pt3glc* petunidin-3-glucoside; *Mv3glc* malvidin-3-glucoside.

Among the numerous families of phenolic compounds, the major one linked to food astringency are the tannins. Tannins’ definition comprises phenolic compounds that “…besides taking part in the usual phenolic reactions have the special ability to precipitate protein”^15^. So, all tannins are phenolic compounds but not all phenolic compounds are tannins. Tannins are usually divided into condensed and hydrolyzable tannins. Condensed tannins are made of structural units of flavanols, namely (epi)catechins and catechin derivatives, that give proanthocyanidins. Hydrolyzable tannins are monosaccharide esters, usually a glucose, of gallic or ellagic acids. In the human diet, condensed tannins are by far the most abundant ones. Several works have shown the relation between flavanols and astringency perception^16–18^. In fact, one of the most widely used compound as a model for astringency studies is epigallocatechin gallate (EGCG)^14^ (Fig. 1), one of the major (astringent) phenolic compound of green tea^19,20^. Epicatechin gallate (ECG) has also been reported with a similar astringency as EGCG^19^.

Regarding food color, the most important phenolic compounds family is the anthocyanins. Widely known for their color portfolio, their colors range from red to blue according to the chemical species present in solution. Depending on the pH, anthocyanins occur in equilibrium between different species, namely flavylium cation (pH < 2–3), hemiketal, quinoidal base (pH 6–8) or chalcones (pH > 9)^21^ . Despite being mainly related to color, some data have also linked anthocyanins to red wine in-mouth organoleptic properties. Sensory analysis data have reported that some anthocyanins have a “mild taste”^22^, and increase astringency, in particular the sub-quality “fine grain”^23,24^. Malvidin-3-glucoside (Mv3glc) (Fig. 1) is the major anthocyanin of red wine. Mv3glc has been reported to interact with salivary proteins through the formation of soluble complexes^23,25^ and it has been also identified as a bitter compound^26^. However, the available data is still very scarce and controversial.

A common feature to most of these studies about the molecular perception of astringency is the use of very simple models, made with model/pure phenolic compounds and proteins. This is, in fact, quite far from what happens in the oral cavity where the intake of phenolic compounds occurs as mixtures and there are several oral constituents present together. Our previous work has evidenced both of these topics regarding procyanidin dimers^27^: that the interaction of some procyanidins is different when they occur as individual compounds or as mixtures, and also a synergy between the different oral constituents on the binding towards procyanidins. Although the previous work has brought a new light about the procyanidin’s interactions in the oral cavity, it raised other key questions: what occurs in mixtures containing other (astringent) phenolic compounds already described by sensory analysis has eliciting different astringent sub-qualities?, what is the contribution of the oral constituents in the binding to different families of phenolic compounds?

In fact, while some flavanol monomers and anthocyanins and have been described as harsh and fine grain (astringent) sensations respectively, to our knowledge, there is a lack of knowledge about their oral interactions, rather than with salivary proteins. Moreover, although astringency can be perceived in different oral tissues, it is so far unknown the possible interactions of phenolic compounds with different oral tissues.

Therefore, this work is focused in deepen the contribution of all the major oral constituents on the oral tissues-(astringent) phenolic compounds interactions. To achieve this, this study was divided in three major aims: i) compare the binding process to different oral cell lines derived from tongue and buccal mucosa; ii) study the contribution of the different oral constituents (oral cells, saliva and mucosal pellicle) on the overall binding of two phenolic extracts rich in flavanols and anthocyanins, respectively; iii) study how (qualitatively and quantitatively) the different phenolic compounds bind to the different oral constituents.

## Results and discussion

### Mucosal pellicle formation using two different cell lines (TR146 and HSC-3)

In this study a model comprising the major constituents of the oral cavity has been used: human oral cell line, human saliva and mucosal pellicle. In order to compare the binding of the phenolic compounds to the different oral cell lines, two models were established: one with a human tongue squamous carcinoma cell line, HSC-3, and one with a human buccal epithelial squamous carcinoma cell line, TR146.

The formation of the mucosal pellicle was achieved by a pre-incubation of the HSC-3 or TRA146 cell line monolayers with mucin 1.0 mg.mL^-1^, followed by an incubation with a pool of human saliva. This methodology has been previously used with success to the formation of the mucosal pellicle with the HSC-3 cell line^12^. So, here it was applied also to the TR146 cell line and only these results will be further discussed in detail^28,29^.

**Figure 2:**
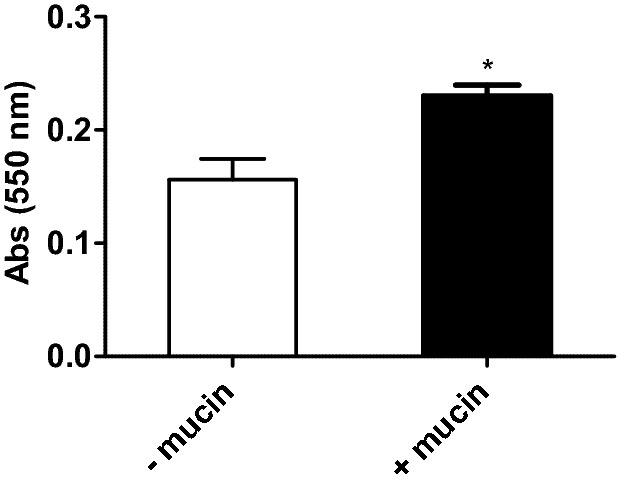
Absorbance at 550 nm of TR146 cell monolayer after the reaction with 1% PA (100 μL) and 80 μL of Schiff-base. These data were obtained for TR146 cell monolayer without (-mucin) and upon incubation with 1.0 mg mL^−1^ mucin (+ mucin) in Hanks buffer. The presented data are the mean of at least three independent experiments; * means are significantly different (P < 0.05).

The first part was to monitor the mucin binding to the TR146 cells which was made by the Periodic acid—Schiff base staining (PAS stain)^30^, a specific stain for glycoproteins. In Fig. 2 it is possible to observe that TR146 cell monolayer alone (- mucin) displayed an absorbance at 550 nm which had a significant increase upon the incubation with mucin (+ mucin). The absorbance of TR146 cell monolayer alone indicates the occurrence of glycoproteins on the epithelial cell surface, which has been already reported for this and other (oral) cell lines^31^. However, the observed increased in the absorbance at 550 nm upon incubation with mucin indicates that this glycoprotein is adsorbed on the TR146 cell surface.

The mucin used in this study (porcine stomach type II mucin) has been already reported to have physical–chemical properties similar to human salivary mucins and has also been a component of simulated saliva fluid^32^. This mucin has a low sialic acid (≤ 1.2%) content (supplier information) being a more neutral mucin. This information was used to choose the PAS stain because it is more useful to recognize also neutral mucins in comparison to other staining techniques (e.g. Alcian blue).

**Figure 3:**
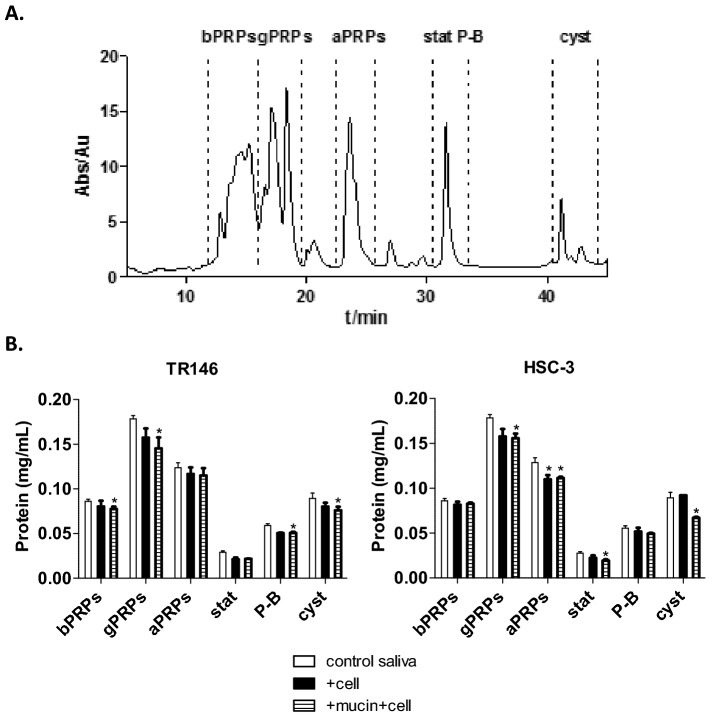
(**A**) Salivary protein profile obtained by HPLC analysis with detection at 214 nm, and identifying the major families eluted along the chromatogram. (**B**) Changes in the concentration (mg mL^−1^) for each family of salivary proteins upon the incubation of human saliva pool with the two cell monolayers (TR146 or HSC-3) before (+ cell) and after pre-incubation with mucin 1.0 mg mL^−1^ (mucin + cell). Data are presented as mean and standard deviation of at least three independent experiments (*, values significantly different from the control saliva, p < 0.05).

After the pre-incubation with mucin, the cell monolayer was incubated with a pool of human saliva. The saliva protein profile was analyzed by HPLC before (control saliva, Fig. 3A) and after the incubation with each cell monolayer alone (TR146 or HSC-3) or with the mucin-cell monolayer (Fig. 3B). The protein profile of human saliva has been previously well characterized and the major families of salivary proteins present after the acidic treatment have been identified^27^. In Figure B it is possible to see a decrease on the concentration of some families of salivary proteins upon the incubation with each cell line alone and for the mucin + cell system. These decreases are attributed to the retention of the protein families to the cell monolayer. In general, these decreases were more significant for gPRPs, aPRPs, P-B peptide and cystatins proteins. However, these decreases were dependent on the cell line. While the retention of gPRPs is quite similar for both TR146 and HSC-3 cell lines, for aPRPs the retention is only significant for the HSC-3 cell line. Also, cystatins show a higher retention for HSC-3 cell line. On the other hand, the bPRPs and P-B peptide retention is only significant for the TR146 cell line. Also, the presence of mucin seems to affect the mucosal pellicle formation significantly only for HSC-3 cell line.

There are several reports on the identification of the salivary proteins that are involved in the formation on the acquired enamel pellicle, but the available data regarding the mucosal pellicle is more limited. Regarding the salivary proteins that form the mucosal pellicle, several proteins (e.g. the proteins CAVI, statherin, cystatin S, and secretory component) have been identified as the main proteins bound to oral epithelial cells. Additionally, histatins, statherin and aPRPs have been identified as precursor proteins to the pellicle formation and able to create a crosslink reaction by oral transglutaminase. Generally, the retained salivary proteins in the developed model are in agreement to the ones found in the literature (for both in vivo and in vitro mucosal pellicle).

Regarding the differences between the two cell lines used in this study (HSC-3 and TR146), besides the different oral localization, another central difference concerns their keratinization. While TR146 cells are described as well-differentiated keratinizing squamous cell carcinoma, HSC-3 cells are described as non- or poor-keratinized squamous cell carcinoma^33,34^. Keratinization has been shown to promote different surface characteristics (microplications or microvilli and pits) of cells from different oral epithelia^35^. In fact, the surface of the epithelia has been shown to be important for the mucosal pellicle formation^36^. The effect of the surface properties (hydrophobic *vs* hydrophilic) in the mucosal pellicle formation and salivary proteins retention has been also previously shown^37^. It has been already anticipated that the mucosal pellicle might differ considerably according to its localization in the oral cavity^38,39^, however no concrete data has been reported yet. To our knowledge this is the first attempt to compare the formation of the mucosal pellicle across the different oral epithelia as well as the salivary proteins present.

At the end, the membrane surface of the epithelia together with the epithelial mucins provide a specialized interface between the saliva and the epithelium that stabilizes the mucosal pellicle. So, it could be expected that changes in one of these components lead to a different ability to form the mucosal pellicle. Our results show small differences on the salivary proteins involved in the mucosal pellicle formation (qualitatively and quantitatively) for the two cell lines, regarding the referred families of salivary proteins analyzed in this study.

### Interaction of different oral constituents with different phenolic compound families

The salivary mucosal pellicle was established with the two different cell lines, from now on referred as HSC3MuSP and TR146MuSP. In this work, both models were used to study the binding process of two phenolic compound extracts, a green tea extract (GTE) and a red wine extract (RWE). It was studied the interaction with each individual oral constituent or when they are present altogether in the HSC3MuSP and TR146MuSP models.

**Figure 4:**
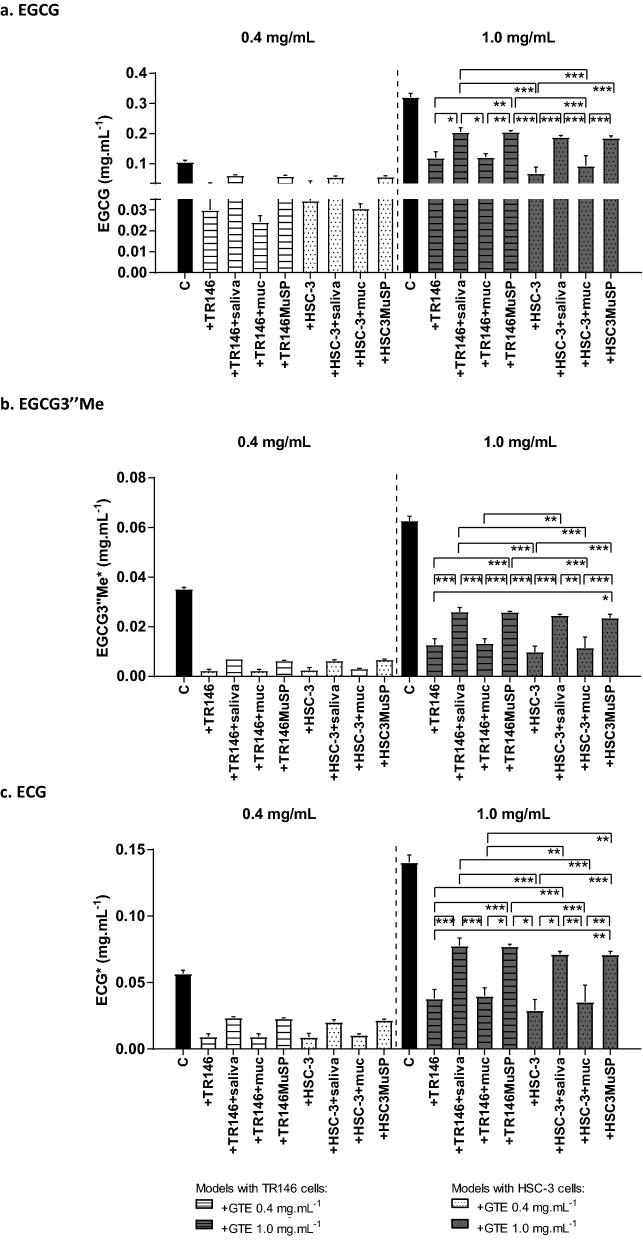
Changes in the concentration of different flavanols (a, EGCG; b, EGCG3″Me; c, ECG) from GTE. The black bar is the concentration in the GTE (control, C); the other bars are the concentrations that were retained in the oral model studied: cell line alone (+ TR146 and + HSC-3), cell line incubated with salivary proteins (+ TR146 + saliva and + HSC-3 + saliva), cell line pre-incubated with 1.0 mg mL^−1^ of mucin (+ TR146 + mucin and + HSC-3 + mucin) and complete oral model with the two cell lines (+ TR146MuSP and + HSC3MuSP). Data are presented as the mean and SEM values for at least three independent experiments; values statistically different are indicated (*p < 0.01, **p < 0.05 and ***p < 0.001). *Express in equivalents of EGCG (mg mL^−1^).

**Figure 5:**
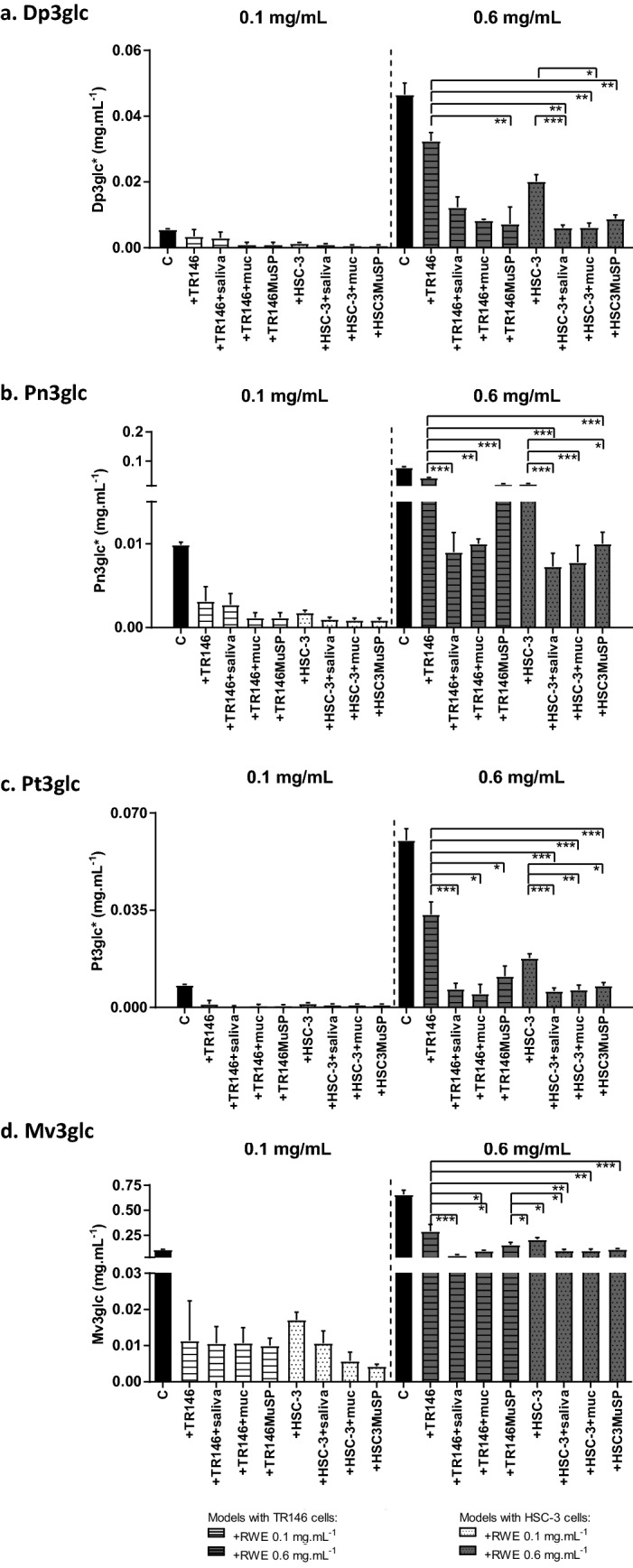
Changes in the concentration of different anthocyanins (**a**, Dp3glc; **b**, Pn3glc; **c**, Pt3glc; **d**, Mv3glc). The black bar is the concentration in the RWE (control, C); the other bars are the concentrations that were retained in the oral model studied: cell line alone (+ TR146 and + HSC-3), cell line incubated with salivary proteins (+ TR146 + saliva and + HSC-3 + saliva), cell line pre-incubated with 1.0 mg.mL^-1^ of mucin (+ TR146 + mucin and + HSC-3 + mucin) and complete oral model with the two cell lines (+ TR146MuSP and + HSC3MuSP). The data are presented as mean and SEM values for at least three independent experiments; values statistically different are indicated (*p < 0.01, **p < 0.05 and ***p < 0.001). *Express in equivalents of mv3glc (mg mL^−1^).

Figure 4 presents the results obtained for the interaction of the individual oral constituents and models with a mixture of flavanols from GTE. The mixture of flavanols was found to have mainly gallocatechin (GC), epigallocatechin (EGC), catechin (C), epigallocatechin (EGCG), procyanidin dimer B2-3′-O-gallate (B2g), epigallocatechin-3-O(3-O-methyl) gallate (EGCG3′’Me), quercetin-rutinoside (Q), epicatechin-3-O-gallate (ECG), kaempferol 3-hexosyldeoxyhesoxide (K), epicatechin-3-O-(4-O-methyl) gallate (ECG3′’Me) (Fig. 1). EGCG and ECG account for 40% of the total weight of the GTE. Twenty six percent of the total weight of the GTE correspond to other compounds (Fig. 1), the minor peaks eluted along the chromatogram. The identifications were made based on literature data^40,41^ and/or comparison with standards, namely for C, ECG, EGCG, Mv3glc. Figure 5 presents the results obtained for the interaction of the referred oral constituents and models with a mixture of anthocyanins from RWE. The mixture of anthocyanins is rich in malvidin-3-glucoside (Mv3glc), which accounts for 79% of the total weight of the RWE. Minor compounds are delphinidin-3-glucoside (Dp3glc), peonidin-3-glucoside (Pn3glc) and petunidin-3-glucoside (Pt3glc) (Fig. 1).

The interactions were made at three concentrations for each extract, 0.4, 0.7 and 1.0 mg.mL^-1^ for the GTE and 0.1, 0.3 and 0.6 mg.mL^-1^ for the RWE. These values are referent to the whole extract. In the final assay, the concentrations of the individual compounds identified before are in the range of the reported concentrations in food matrices^42,43^. The extracts were analyzed by HPLC before (control, C) and after the interaction with the different oral constituents: monolayer of each cell line (+ TR146 or + HSC-3), monolayer of each cell line incubated with human saliva (+ TR146 + saliva or + HSC-3 + saliva), monolayer of each cell line pre-incubated with mucin (+ TR146 + mucin or + HSC-3 + mucin) and the mucosal pellicle model (+ TR146MuSP or + HSC3MuSP). The concentration (mg.mL^-1^) of the phenolic compounds retained in each case (the bound concentration) was determined by subtracting the concentration that remained in solution after incubation with each experimental condition to the initial concentration (control condition, C).

Figure 4 displays the data only for the flavanols of the GTE (at 0.4 and 1.0 mg.mL^-1^) that had the highest interaction (EGCG, EGCG3″Me, ECG). The data obtained for these flavanols at GTE 0.7 mg.mL^-1^ as well as for the other compounds of the GTE (C, GC, EGC, B2g, Q, K and ECG3″Me) are provided as Supplementary Information (Figure S1). Furthermore, the other minor compounds that correspond to 26% of the GTE were not considered for analysis because they remain equal before and after the interactions. Figure 5 shows the data for the anthocyanins of the RWE (at 0.1 and 0.6 mg.mL^-1^). The data obtained for RWE 0.3 mg.mL^-1^ are provided as Supplementary Information (Figure S2).

In general, for all the compounds it is possible to observe an increase of the retained quantity along with the increases in the applied concentrations. This is highly evident for EGCG and ECG upon GTE incubation and for mv3glc upon RWE incubation.

For all the compounds, looking at a specific concentration, it is possible to observe a similar retained concentration between the TR146 and HSC-3 cell monolayers (+ TR146 and + HSC-3 conditions, respectively). This also occurs in the presence of mucin (+ TR146 + muc and + HSC-3 + muc). So, it seems that the interaction of these compounds does not depend on the type of epithelial cell line. As referred previously, the major differences between these cell lines is their oral localization and their keratinization (HSC-3 derive from human tongue and are non-keratinized; TR146 derive from human buccal and are keratinized). These differences seem to not affect neither quantitatively neither qualitatively the binding ability of the phenolic compounds studied.

In a recent work with lipid membrane systems bearing different contents in cholesterol, it was found that EGCG showed a higher interaction to the system with low ratio in cholesterol. These systems have been assembled to mimic those epithelial environments found in different oral mucosa regions and tongue^44^. The results suggested a higher interaction of EGCG with the more hydrophilic buccal mucosa, floor of the mouth and gingiva regions rather than with the more hydrophobic regions such as palate and tongue. This was not observed in this work, but there is no data about the cholesterol content of the cell lines used herein.

Recently, the impressive ability of the human tongue to differentiate very small differences in surface texture regarding astringency perception has been reported. At the light of the results obtained here, it can be inferred that this ability is not probably intrinsically related to the tongue epithelia itself, since the buccal epithelium has a similar behavior, but is probably related to the ultrastructure of the tongue within the buried epidermal layers and nerve afferents of the tongue. In this light, one possible hypothesis is that these flavanols-oral cells or the salivary proteins-flavanols-oral cells interactions can activate mechanoreceptors, buried within the tongue epidermal layers. In fact, the activation of the trigeminal nerve has been already proved for EGCG^14^.

A tendency observed for the interaction of the flavanols present in the GTE is a higher interaction with TR146MuSP and HSC3MuSP models in comparison to TR146 and HSC-3 cell monolayers alone, respectively. This increase is evident for EGCG, EGCG3″Me and ECG, in particular at the highest concentration. Once again, also in these mucosal pellicle models, the interaction seems independent of the cell line since TR146MuSP binds the same concentration of phenolic compound as HSC3MuSP.

However, for the anthocyanins in the RWE it was observed an opposite behavior with a lower interaction with TR146MuSP and HSC3MuSP models in comparison to the cell lines alone. Although this decrease occurs for all the studied anthocyanins and concentrations, it is only significant for the highest concentration of RWE (0.6 mg mL^−1^).

Another common trait regarding the interaction of flavanols is an increase on the interaction for all the oral models that present salivary proteins (+ TR146 + saliva, + HSC-3 + saliva, + TR146MuSP and + HSC3MuSP). Once again, this effect is independent of the cell line present. On the other hand, for the interaction of anthocyanins the presence of salivary proteins and/or mucin seems to significantly reduce the retained concentration, especially for the highest studied concentration. While the interaction of the flavanols seems somehow to be driven by the presence of salivary proteins, for anthocyanins the presence of salivary proteins or mucin seems to protect their interaction with the oral constituents.

Ultimately, the only common trend between the interactions of flavanols and anthocyanins with the oral constituents is the similar interaction between the two oral cell lines. Thereafter, the influence of the oral constituents is different for flavanols and anthocyanins. On one hand, the presence of saliva and/or mucin seems to increase the interaction of flavanols to the oral models, while on the other hand the presence of these oral constituents seems to decrease the interaction of anthocyanins.

### Interaction of phenolic compounds (structure- and concentration-interaction relationships) with the oral constituents

**Figure 6:**
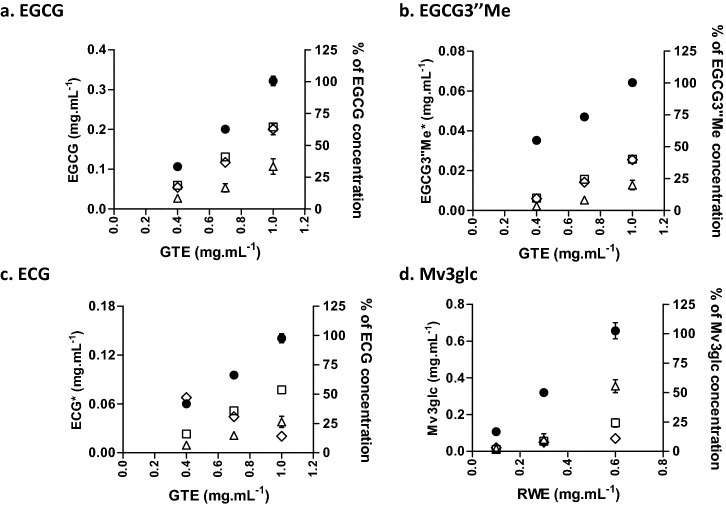
Changes in the concentration of each phenolic compound. The black dot (filled circle) is the initial concentration of the compound in the extract. The left *y-axis* presents the concentration in mg mL^−1^ while the right *y-axis* presents the normalization considering the highest concentration of the compound as 100%. The other marks are the retained concentration for the different model systems: interaction with TR146 cell monolayer (triangle), mucosal pellicle model (TR146MuSP, open rectangle) and human saliva (diamond symbol). The binding experiments were made for three concentrations of the GTE, 0.4, 0.7 and 1.0 mg.mL^-1^ and three concentrations of the RWE, 0.1, 0.3 and 0.6 mg mL^−1^. Data are presented as the mean and SEM of at least three independent experiments. *Express in equivalents of EGCG (mg mL^−1^).

Figure 6 presents the binding of the flavanols of the GTE that had the highest interaction (EGCG, EGCG’’3Me and ECG) upon the interaction with TR146 cell monolayer, with the TR146MuSP model and with human saliva. The results obtained for the models with HSC-3 cell line were similar (Supplementary Information, Figure S3).

EGCG was the compound with the highest binding to the TR146 cell monolayer and TR146MuSP model. It is possible to observe this by normalizing the concentration effect, i.e., by normalizing the differences on the concentration for the different compounds. This was achieved considering the highest concentration for each compound as 100% (right *y-axis)*. Due to the concentration effect, a compound that occurs in higher concentration will always be able to interact in higher concentration. To compare the reactivity of the different compounds, the real concentration that interacted is not so significant. But if the concentration effect is normalized, it will allow to determine the reactivity toward an oral constituent. For the highest concentration of EGCG, 65% of its initial concentration interacted with TR146MuSP against 30% that interacted with TR146 cell monolayer. For the highest concentration of ECG, 52% of its initial concentration interacted with TR146MuSP against 25% that interacted with TR146 cell monolayer. These percentage values are even lower for the EGCG3″Me. So, EGCG3″Me had a significantly lower interaction evidencing that the addition of a methyl group in the structure of EGCG had a dramatic effect on reducing the ability to interact with the used models. These results could also support the importance of the galloyl group as a major molecular site for the interactions with the oral constituents, has previously shown for model lipid membranes^44,45^. The lower interaction of ECG to TR146 in comparison to EGCG is in agreement with a previous work^4^. Moreover, a recent work that assembled lipid membrane models bearing different contents in cholesterol, showed a similar interaction of ECG and EGCG to the models with the lowest content in cholesterol^44^. On the other hand, the ECG has been found to have a higher interaction than EGCG upon the interaction with lipid (no-cholesterol) membrane models^45,46^. Therefore, the differences between the ECG and EGCG interactions observed here could be due in part to the different content of cholesterol of the studied cell lines.

A critical property of phenolic compounds that could affect their reactivity and interactions is their self-association. Above a threshold concentration, the critical micellar concentration (CMC), the self-association of their molecules leads to the formation of colloidal particles that can reach hundreds of nanometers. The CMC of these flavanols has been reported to be 6.0 mM (2.7 mg.mL^-1^) for EGCG ^47^ and much lower for ECG 2.8 mM (1.2 mg.mL^-1^) ^48^. Moreover, in complex systems like GTE it is expected the occurrence of co-aggregation with other flavanols affecting their CMC and consequently their overall interactions ^49^.

Regarding the structure-interaction relationship toward salivary proteins, in general, it was observed a similar behavior as for the TR146MuSP model, with the exception of the ECG. The highest ECG interaction with salivary proteins occurred for the ECG lowest concentration (0.4 GTE mg.mL^-1^) with almost all available ECG interacting. Thereafter, the increases in the ECG concentration decreased the interaction with salivary proteins. The possible explanations could be also based on the previously referred self-association and interaction with the other phenolic compounds present in the mixture. Another possible explanation occurs in the light of the postulated mechanism for polyphenol-protein interaction^50^. For a fixed protein concentration there is an optimum phenolic compound concentration at which there is a maximum of interaction (plateau of maximum interaction). Before and after this plateau there is a decrease on the interaction^50^. For ECG, this maximum of interaction could be equal or lower than 0.06 mg.mL^-1^, since higher ECG concentrations lead to a lower interaction. Based on this, it could be inferred that ECG presented a higher interaction with salivary proteins than EGCG. In fact, for the interaction with 0.06 mg.mL^-1^ of ECG (at 0.4 mg.mL^-1^ of GTE), the ECG was completely bound to salivary proteins while at a similar concentration of EGCG it was not the case. The higher interaction of ECG in comparison to EGCG has been previously observed for the interaction with a proline-rich peptide by ESI–MS studies^51^.

For the interactions with TR146 cell line and with the TR146MuSP model it was observed an increased binding of each compound along with their initial concentration. A similar trend was observed for the models with HSC-3 cell line (Supplementary Information, Figure S3).

Looking at the EGCG interaction, it is possible to observe that the TR146MuSP model bound near 0.2 mg.mL^-1^ of EGCG upon the incubation with 0.33 mg.mL^-1^ of this compound (GTE 1.0 mg.mL^-1^). Therefore, it was expected that during the incubation with 0.7 mg.mL^-1^ of GTE extract (that presents 0.2 mg.mL^-1^ of EGCG), all EGCG would be completely depleted, which is not the case. This behavior was similar also for EGCG3′’Me and ECG.

So, it seems that the molecules of these flavanols attain an equilibrium at a specific concentration between the unbound and bound molecules, and therefore there is not a complete interaction with all the molecules available, even for the lowest concentration. The previously referred self-association of these compounds could also justify this equilibrium^51^.

The significantly lower interaction observed for some of the other flavanols, namely EGC and catechin (data not shown), both with the mucosal pellicle models, with salivary proteins as well as with individual cell lines, has been previously reported with lipid membrane models^45^ and other peptides/proteins^51^. These lower interactions have been explained by the lack of a galloyl moiety, whose importance on these interactions has been reinforced by these results. It is also interesting the absence of interaction of quercetin and kaempferol with any oral cell model studied here, despite both compounds have been previously reported to interact with lipid membrane models^52^. Some authors observed that their interaction was more pronounced in the absence of cholesterol, which is not the case in the cell membrane of human cell lines, usually rich in cholesterol. This could justify the absence of interaction with the cell line models. Also, quercetin has been reported to interact with salivary proteins^53^. These authors studied the interaction with salivary proteins present altogether but were focused only on soluble complexes. The approach used in our study only allows to identify and monitor interactions that lead to the formation of insoluble precipitates which are removed by centrifugation. All the interactions that lead to soluble complexes could not be monitored by this approach. This could explain the contrasting results for quercetin compound.

Figure 6 presents also the data for the interaction of mv3glc with TR146 cell monolayer, with the TR146MuSP model and with human saliva. For the other anthocyanins the trend was similar as well as for HSC-3 cell line (Supplementary Information, Figure S3). For the anthocyanins, the interaction ability between the several compounds was similar.

Looking at the normalized concentrations, all the anthocyanins had an interaction of 55% of the available concentration toward TR146 cell monolayer. Regarding the TR146MuSP model all anthocyanins, except Pt3glc, showed an interaction of 25%. Pt3glc presented a much lower interaction. It is interesting that these compounds display a high similarity of interaction toward a particular oral model and they also share a very similar molecular structure (Fig. 1). It seems that opposing to flavanols, small changes on anthocyanin structures do not change significantly their ability to interact with cells or with salivary proteins.

Regarding the concentration effect, an increase in the binding of Mv3glc along with its initial concentration was only significant for the TR146 cell monolayer. For the TR146MuSP model the Mv3glc concentration effect was very and for the other anthocyanins was practically negligible for the TR146MuSP model and for the interaction with salivary proteins.

However, regarding the concentration effect there is a similarity between anthocyanins and flavanols. Looking at the interaction of anthocyanins with TR146 cell monolayer and with the TR146MuSP model, they also seem to attain an equilibrium between the unbound and bound molecules, and therefore there is not a complete interaction with all the available molecules. This is not strange since anthocyanins are also known for their self-association^54,55^ which in this case besides being affected by their concentration is also affected by the pH. In this study the experiments were all done at the same pH, pH 6.5. At this pH anthocyanins are essentially present in its colorless hemiketal form (p*K’*_*h*_ 2–3) in equilibrium with the flavylium cation and quinoinidal base forms (pK_a1_ ~ 4). Therefore, it is expected that at this pH the major form involved in the interaction was the hemiketal form. However, there is no data about which chemical species of anthocyanins could have a higher interaction with salivary proteins or with cells membrane.

To our knowledge there is not any report about the interaction of mv3glc with lipid membrane models. The interaction of some anthocyanins (pelargonidin-3-glucoside and cyanidin-3-galactoside) with simple and more complex lipid membrane models has been previously reported^56^. However, most of these studies were focused on several cyanidin derivatives^57,58^. An extensive study about the relationship between structure-lipid membrane interactions of cyanidin derivatives has shown that glycosylation number and the presence of aromatic acid attached to sugars affected the membrane binding and properties^59^. Most of the reported studies do not gather information about the interaction of the different chemical species of anthocyanins in equilibria. The tiny structural differences among the studied anthocyanins here are essentially the presence of methyl and/or hydroxyl groups, which in the case of the cyanidin derivatives were not significant for changes in the interaction with the lipid membrane models. In addition, as previously mentioned, these structural changes here do not seem significant for malvidin derivatives.

### Salivary proteins involved in the interaction with phenolic compounds in the different oral models

**Figure 7:**
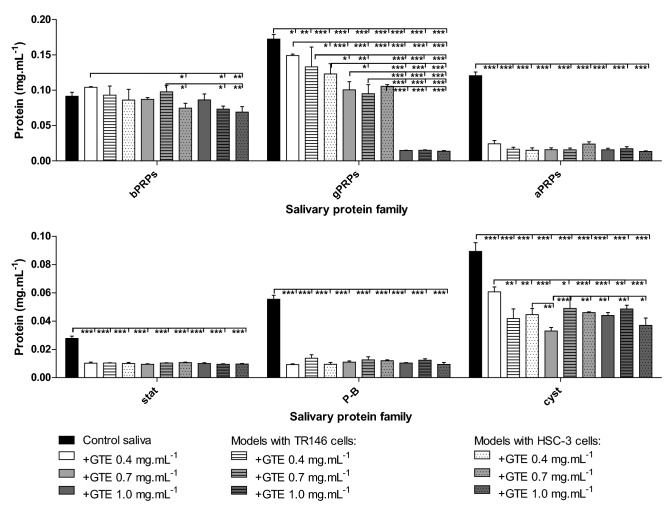
Changes in the concentration of salivary protein families before (control saliva) and after the interaction with GTE alone, and after the interaction in presence of the two oral mucosal pellicle models (TR146MuSP and HSC3MuSP). Data are presented as mean and SEM of at least three independent experiments. Values statistically different are indicated (*p < 0.01, **p < 0.05 and ***p < 0.001).

**Figure 8:**
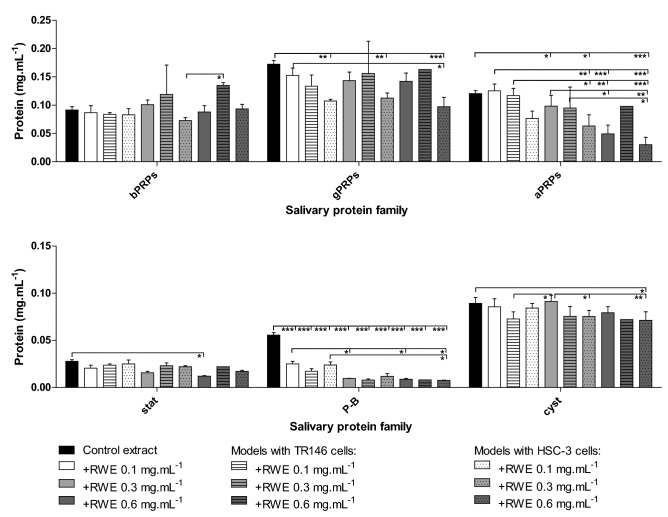
Changes in the concentration of salivary protein families before (control saliva) and after the interaction with RWE alone, and after the interaction in presence of the two oral mucosal pellicle models (TR146MuSP and HSC3MuSP). Data are presented as mean and standard deviation of at least three independent experiments. Values statistically different are indicated (*p < 0.01, **p < 0.05 and ***p < 0.001).

The salivary protein profile of the human saliva pool has been previously well characterized by HPLC^27^. The changes on the profile of the salivary protein families mostly linked to astringency perception were followed upon the interaction with the GTE (Fig. 7) and with the RWE (Fig. 8). These changes were followed upon the interaction of each extract with human saliva alone and upon the interaction with the developed mucosal pellicle models (TR146MuSP and HSC3MuSP). The data obtained for the interaction with the saliva + TR146 and saliva + HSC-3 (data not shown) were similar to the ones obtained for the mucosal pellicle models.

From Fig. 7 it is possible to observe that the interaction with flavanols, regardless of the concentration used, lead to a total depletion of the salivary proteins aPRPs, statherin and P-B peptide. This means that these salivary proteins were completely precipitated by flavanols, even knowing that it was not observed a total depletion of the available concentration of the flavanols. However, for bPRPs and gPRPs it is possible to observe the effect of flavanols concentration. There is an increase of these proteins’ depletion along with the GTE concentration. For most cases, the depletion of salivary proteins is quite similar among the different oral models. However, in some cases it seems that the interaction with HSC3MuSP model leads to a higher depletion of some families in comparison to the saliva alone, namely for cystatins and bPRPs. This is especially evident for the highest GTE concentration and also for gPRPs at the lowest GTE concentration.

The behavior is quite different for the interaction with RWE (Fig. 8), as in this case there is only a significant depletion of P-B peptide. Increasing the RWE concentration also leads to precipitation of other families, such as aPRPs and gPRPs, however these interactions do not lead to a total depletion of these proteins, at least for the RWE concentrations studied here. In a similar way to the flavanols, for some cases the depletion of the proteins was higher for the HSC3MuSP model in comparison to the saliva alone. This was evident for the gPRPs and bPRPs. At the end, although no differences between the two cell lines have been observed for the interaction with the phenolic compounds, regarding the precipitated salivary proteins there seems to be a difference with the mucosal pellicle based on HSC-3 cells presenting a slightly higher precipitation of salivary proteins.

Flavanols and anthocyanins occur frequently in common foodstuffs. From our results, it can be conjectured that the flavanols present in the food matrix seem to interact more than anthocyanins. In summary, this work showed that when all the oral constituents occur together, the oral interactions of flavanols seems to be driven by salivary proteins, leading to their significant depletion. On the other hand, oral cells were shown for the first time to be the oral constituent with the major interaction toward anthocyanins. This could be an important feature when there is a significant depletion of the human saliva upon food intake.

This work also gathered first-time evidences that the studied flavanols and anthocyanins seem to bind in the same way to two oral cell lines, one derived from human tongue and the other one derived from human buccal mucosa.

Throughout the literature, the flavanols present in GTE are referred as some of the most astringent and harsh compounds while anthocyanins have been linked to soft sub-qualities of astringency.

Overall, the obtained results suggest that the engagement of different oral constituents into the interactions could be related to the perception of different astringency sub-qualities. Moreover, the similar interaction displayed by different epithelial oral cells, suggested for the first time that other structures of the tongue, such as the buried mechanoreceptors, could also be the source of specific astringent sensations.

Ongoing studies are focused on determining the specificity of the developed models regarding the interaction with non-astringent phenolic compounds, on determining if the order of interaction could be an important factor for these oral interactions/astringency perceptions, as well as to find ways to modulate these interactions.

## Materials and methods

### Saliva isolation and analysis

Human saliva was isolated from several healthy non-smokers’ volunteers (males and females, ages between 23 and 40 years old). The saliva from each volunteer was pooled together to gather a whole representative human saliva. The pool of saliva was treated with 0.1% TFA to partially inhibit proteases activity and precipitate several high molecular weight proteins not possible to be analyzed by HPLC^27^. After this treatment, saliva was centrifuged at 4 °C for 5 min at 10,500 rpm. The supernatant was recovered, aliquoted and kept at -80 °C until use. Several chemical-physical parameters of the isolated saliva are routinely analyzed, namely the ionic strength, the protein concentration, and salivary protein profile. The ionic strength was analyzed by a conductimeter (WTM Inolab 740) and it was determined to be 0.7 S/m. The protein concentration was assessed using the Bradford assay. 15 µL of saliva were added to 150 µL of the Bradford reagent (Coomassie brilliant Blue G-250, Sigma). The samples were mixed and kept for 10 min at room temperature in the dark. A calibration curve was made with BSA (0.0, 0.1, 0.5, 0.8, 1.1, 1.5 mg.mL^-1^) in the same way as described. After, the samples were transferred to a 96 well plate and the absorption at 595 nm was read in a plate reader (BioTek Instruments, Winooski, VT, USA). The protein concentration was determined to be 0.68 mg.mL^-1^. The salivary protein profile was analyzed by a HPLC equipped with a Kinesis Telos reversed-phase C8 column (150 × 2.1 mm, 5 µm). The solvents used were solvent A, 0.2% aqueous TFA and solvent B, 0.2% TFA in ACN/water 80/20 (v/v). The elution program was 11% to 45% of B during 50 min at a flow of 0.5 mL.min^-1^. Detection was carried out at 214 nm.

The salivary protein family present in the chromatogram have been previously identified by a proteomic approach^27,60^ .

Prior to saliva isolation all the participants were gave an informed consent form to sign. The consent form was obtained for all participants. The study was conducted according to the Declaration of Helsinki and was approved by the Ethics Committee of University of Porto (CES183/18).

### Green tea extract (GTE) rich in flavanols

A GTE rich in flavanols was obtained from twenty grams of dried green tea leaves of *Camellia sinensis* from Portuguese origin (Gorreana, Portugal). The leaves were grinded and extracted three times with 500 mL of 80% (v/v) ethanol for 1 h at room temperature and under argon atmosphere. Between extractions, the slurry mixtures were centrifuged at 12,000×*g* for 20 min at 4 °C and the resulting supernatants combined. The organic solvent was then removed using a rotary evaporator under reduced pressure resulting in a highly dense green residue. This residue was subsequently extracted three times with an equal volume of chloroform to remove methylxanthines and chlorophylls. Then, ethyl acetate was added in the same proportion of aqueous solution to extract green tea flavanols, being this step repeated three additional times. After ethyl acetate evaporation, the resulting flavanols-rich residue was loaded into an Oasis HLB 35 cc Vac cartridge from Waters and washed with 5% acetonitrile/1% acetic acid to remove sugars, organic acids, and other polar compounds. The phenolic fraction was subsequently recovered by eluting the cartridge with an acetonitrile/methanol mixture (50:50, v/v). Later, the organic solvent was evaporated and the resulting green tea extract (GTE) freeze-dried. The GTE was routinely analyzed on a Jasco LC-4000 HPLC system equipped with an Agilent Poroshell 120, C18 reverse-phase column (250 × 4.6 mm, 2.7 μm particle diameter). The solvents were A: 0.1% formic acid in water and B, 0.1% formic acid in acetonitrile; linear gradient (0 min, 10% eluent B to 50 min, 28% eluent B); flow rate of 0.5 mL min^−1^ and detection at 280 nm, using a Photo diode array detector (MD-4010). To identify the compounds present, the GTE was analyzed by LC–MS (Finnigan DECA XP PLUS). The compounds were identified based on the mass spectra data, retention time of standards and/or comparison with the literature (Fig. 1).

In order to determine the concentration of the compounds, a calibration curve was made with purchased EGCG (Biopurify). The calibration curve was established for an EGCG concentration range of 0.025 to 1.0 mg.mL^-1^ and was determined to be x = (y + 744,946)/3 × 10^+07^.

### Red wine extract (RWE) rich in anthocyanins

A RWE rich in anthocyanins was obtained from concentrated red wine as described previously^61^. Briefly, 50 L were concentrated by nanofiltration and the ethanol was then evaporated. Sugars were removed by C18 reverse gel loaded on a Buchner funnel and then the obtained wine was lyophilized. Five grams of the lyophilized powder were extracted with ethyl acetate to remove the non-anthocyanins phenolic compounds. The aqueous layer was then applied to a TSK Toyopearl gel HW-40(S) and then the extract rich in anthocyanin 3-monoglucosides was eluted with 10% aqueous acidified methanol. The methanol was evaporated and the aqueous phase concentrated in a rotary evaporator under reduced pressure at 30 °C. The resulting extract (RWE) was then freeze-dried. The RWE was routinely analyzed on a Jasco LC-4000 HPLC system equipped with an Agilent Poroshell 120, C18 reverse-phase column (250 × 4.6 mm, 2.7 μm particle diameter). The solvents were A: 10% aqueous formic acid and eluent B, 10% formic acid in 30% acetonitrile; linear gradient (0 min, 25% eluent B to 35 min, 65% eluent B); flow rate of 0.5 mL·min^−1^ and detection at 520 nm, using a Photo diode array detector (MD-4010). The RWE was analyzed by LC–MS and the compounds were identified based on the retention time, comparison to standards and/or comparison with the literature (Fig. 1)^61^.

In order to determine the concentration of the compounds, a calibration curve was made with pure Mv3glc. The calibration curve was established for a Mv3glc concentration range of 0.05 to 1.0 mg mL^−1^ and was determined to be x = (y-10936)/4 × 10^+06^.

### Cell culture conditions

Two epithelial-like squamous cell carcinoma cell lines were used in this study: HSC-3 cell line derived from human oral tongue and TR146 derived from buccal mucosa. HSC-3 cells were routinely grown up in DMEM-high glucose supplemented with 1% Ala-Gln (200 mM solution) (Sigma-Aldrich, Darmstadt, Germany) and TR146 cells in HAMS F12 containing 2 mM Glutamine. Additionally, both media were supplemented with 10% Fetal Bovine Serum heat inactivated (Biowest, Nuaillé, France) and with 1% antibiotic/antimycotic solution (100 U/mL of penicillin, 100 μg/mL of streptomycin, and 0.25 μg/mL of amphotericin B) (Sigma). Cells were maintained under conditions of 5% CO_2_ in air at 37 °C in a humidified atmosphere and were dissociated with trypsin (0.25% (w/v) trypsin-EDTA4Na) at 80–90% confluence.

### Oral model development: mucin adhesion and salivary proteins binding

The development of the oral model has been previously reported for the HSC-3 cells^12^. In this study a similar approach was used to development a similar model with TR146 cells. Briefly, the first part consisted on mucin adhesion to the TR146 cells. TR146 cells were seeded into 96 well flat-bottomed tissue culture plates at a density of 2.0–5.0 × 10^4^ cells/well and grown until 90–100% confluence to be used in an assay. Prior to mucin adhesion, the growth medium was removed and the cell monolayers were washed twice with warmed PBS, pH 7.6. A stock solution of mucin from porcine stomach (20 mg.mL^-1^) was made in NaOH (1 M) and was then diluted with HANKS buffer to obtain a final mucin solution at a 1.0 mg.mL^-1^. Final pH was adjusted to 7.0 30 μL/well was applied and incubated during 1 h in the same conditions used for cell growth. After, the mucin solution was aspirated and the Periodic Acid-Schiff base (PAS) coloration for glycoprotein detection was performed with some modifications^30,62^. To each well, 100μL of 1% periodic acid dissolved in 3% acetic acid was added and the plate was incubated 30 min, at room temperature in the dark. Then the periodic acid was removed, 80μL of the Schiff base (Sigma) was added and the plate was incubated 1 h, at room temperature in the dark. After the incubation, the Schiff base was carefully removed, the wells were washed and 50 μL of DMSO (Sigma-Aldrich, Darmstadt, Germany) was added. The plate was gently shake and the absorbance was measured at 550 nm in a plate reader (BioTek Instruments, Winooski, VT, USA) in order to confirm the adhesion of mucin to the TR146 cell monolayer.

The mucosal pellicle formation on the TR146 cells was done in a similar way as described previously for HSC-3 cells^12^. Mucin adhesion to a TR146 cell monolayer was made as described above to 48 of the 96 wells. The other half remained with growth medium. After 1 h incubation, the half of the plate without mucin was washed twice with warmed PBS, pH 7.6 and mucin was aspirated from the other wells. Then 30 μL/well of the isolated saliva (with the pH adjusted to 6.5) was added to 72 wells. To the left 24 wells it was added 30 μL of aqueous buffer (pH 6.5) instead of saliva (control wells). The plate was incubated 2 h in the same conditions used for cell growth. At the same time, it was prepared an exactly equal plate but without cells. After the respective incubation time, the solutions were collected, centrifuged at 13,400 rpm for 5 min and the supernatants were recovered. During these manipulations, microtubes were always kept on ice and immediately frozen at − 80 °C until analyzed. At the end, several aliquots were gathered corresponding to saliva, mucin + saliva, saliva + TR146 and mucin + saliva + TR146 conditions. The salivary protein profile changes were analyzed by HPLC as described before.

### Interaction of the phenolic extract with the oral model with TR146 and HSC-3 cells

Similarly to the assay described above, TR146 or HSC-3 cells were seeded into 96 well flat-bottomed tissue culture plates and grown until reaching confluence. Half of the 96 well plates were washed twice with PBS, pH 7.6, to remove residual growth medium. 30 μL of the mucin solution (1 mg mL^−1^, pH 7) was added and the plate was incubated for 1 h. A lot of 48 wells were maintained without mucin wittingly. After 1 h, these wells without mucin were washed twice with PBS, pH 7.6 and the mucin was carefully aspirated from the other wells. 30 μL of human saliva (with the pH adjusted to 6.5) was added to 72 wells (with and without mucin pre-incubation). To some wells (24) saliva was replaced by aqueous buffer (pH 6.5). An exactly equal 96 well plate was prepared without cells. After 2 h of incubation, 15 μL of three different stock concentrations of each extract (1.2, 2.1 and 3.0 mg.mL^-1^ for the GTE; 0.3, 1.2 and 1.8 mg.mL^-1^ for the RWE) were added to different wells and to both plates (with and without cells) and the plates were incubated for 15 min at 37 °C. After this time, the solutions were collected, centrifuged at 13,400 rpm for 5 min and the supernatants were recovered. During these manipulations, microtubes were always kept on ice and immediately frozen at − 80 °C until analyzed. All the supernatants of the RWE were acidulated with 2%HCl previously to being frozen. Supernatants were analyzed by HPLC to determine the changes in the phenolic compounds profile and the changes in the salivary protein profile. The HPLC conditions for each analysis are the ones referred previously.

At the end, six experimental conditions were set for each extract concentration and each cell line, covering all the control conditions and all the oral constituents: + Extract, + Extract + cell, + Extract + saliva, + Extract + saliva + cell, + Extract + mucin + cell, and + Extract + mucin + saliva + cell. The concentration of the phenolic compounds in each experimental condition was assessed using the respective established calibration curve.

### Viability assay

The MTT assay was made in the 96 well flat-bottomed tissue culture plates for the referred cell monolayers as described previously^12^. Briefly, after recover of each experimental condition, cells were washed with PBS, pH 7.6 and 100 μL of the MTT (Himedia Laboratories, Einhausen, Germany) solution (0.5 mg.mL^-1^ in the culture medium) was added and incubated for 30 min under normal growth conditions. The solution was discarded and the wells washed with PBS, pH 7.6. Then, 100 μL of DMSO was added and the absorbance measured at 540 nm with a microplate reader (Molecular Devices, LLC., San Jose, USA). Data are presented as Supplementary Data (Fig. S4).

### Statistical analysis

All assays were performed at least in triplicate independent experiments, in separate days and replicates within the same assay. The mean values and SEM were evaluated using analysis of variance (ANOVA) followed by Bonferroni post-hoc test; all statistical data were processed using GraphPad Prism version 5.0 for Windows (GraphPad Software, San Diego, CA; www.graphpad.com).

## Supplementary information

Supplementary file1 (DOCX 411 kb)
