# Supplementary material for: Oral interactions between a green tea flavanol extract and red wine anthocyanin extract using a new cell-based model: insights on the effect of different oral epithelia
Source: Sci Rep. 2020 Jul 28;10:12638. doi: 10.1038/s41598-020-69531-9 (PMC7387539; doi:10.1038/s41598-020-69531-9)
Supplement: Supplementary file 1 — Supplementary file1 (DOCX 411 kb) [file 41598_2020_69531_MOESM1_ESM.docx]

**Oral interactions between a green tea flavanol extract and red wine anthocyanin extract using a new cell-based model: insights on the effect of different oral epithelia**

Susana Soares*^1^, Sónia Soares^1^, Elsa Brandão^1^, Carlos Guerreiro^1^, Nuno Mateus^1^, Victor de Freitas^1^

LAQV/REQUIMTE, Faculdade de Ciências da Universidade do Porto, Rua do Campo Alegre, 687, 4169-007, Portugal

*susana.soares@fc.up.pt

**Supplementary Data**

**Figure S1.**

**A.**

**B.**

**Figure S1.** **Changes in the concentration of different compounds** (GC, gallocatechin; B, EGC, epigallocatechin; B2g, procyanidin B2-3-O’-gallate; Q, quercetin; K, kaempferol; ECG3’’Me, epicatechin-3-O-(4-O-methyl) gallate) **from GTE that: (A) were not retained and (B) were retained in the oral model studied**: C (black bars), in GTE, cell line alone (+TR146 and +HSC-3), cell line incubated with salivary proteins (+TR146+saliva and +HSC-3+saliva), cell line pre-incubated with 1.0 mg.mL^-1^ of mucin (+TR146+mucin and +HSC-3+mucin) and complete oral model with the two cell lines (+TR146MuSP and +HSC3MuSP). Data are presented as the mean and SEM values for at least three independent experiments. *Express in equivalents of EGCG (mg.mL^-1^).

**Figure S2.**

**Figure S2.** **Changes in the concentration of different compounds** (Dp3glc, delphinidin-3-glucoside; Pn3glc, peonidin-3-glucoside; Pt3glc, petunidin-3-glucoside; Mv3glc, malvidin-3-glucoside) **from RWE that were applied to the oral model studied**: C (black bars), in RWE, cell line alone (+TR146 and +HSC-3), cell line incubated with salivary proteins (+TR146+saliva and +HSC-3+saliva), cell line pre-incubated with 1.0 mg.mL^-1^ of mucin (+TR146+mucin and +HSC-3+mucin) and complete oral model with the two cell lines (+TR146MuSP and +HSC3MuSP). Data are presented as the mean and SEM values for at least three independent experiments. *Express in equivalents of Mv3glc (mg.mL^-1^).

**Figure S3.**

**A.**

B.

C.

**Figure S3.** Changes in the concentration of the referred phenolic compounds from the extract before (⚫) and retained in the model system after: interaction with cell monolayer (△, A. and C. HSC-3 cell line and B. TR146 cell line), mucosal pellicle model (□, HSC3MuSP for A. and C.; TR146MuSP for B.) and human saliva (◇). The left *y-axis* is the concentration (mg.mL^-1^) based on specific calibration curves (experimental section) and the right *y-axis* is the normalization considering the highest concentration of each compound as 100%. The binding experiments were made for three concentrations of the GTE, 0.4, 0.7 and 1.0 mg.mL^-1^ and three concentrations of the RWE, 0.1, 0.3 and 0.6 mg.mL^-1^. Data are presented as the mean and SEM of at least three independent experiments. *Express in equivalents of EGCG (mg.mL^-1^); **Express in equivalents of Mv3glc (mg.mL^-1^).

**Figure S4.**

**Figure S4.** **Percentage of viable cells determined by the MTT assay, using the respective cell line alone (HSC-3 or TR146) as control (100%).** Viability was determined for the following conditions: cell line alone (TR146 or HSC3), cell line incubated with salivary proteins (TR146SP and HSC-3SP), cell line pre-incubated with 1.0 mg.mL^-1^ of mucin (TR146Mu and HSC3Mu) and complete oral model with the two cell lines (TR146MuSP and HSC3MuSP) in absence and presence of the green tea extract (GTE) and red wine extract (RWE).
